# *Cis*-regulatory *PLETHORA* promoter elements directing root and nodule expression are conserved between *Arabidopsis thaliana* and *Medicago truncatula*

**DOI:** 10.1080/15592324.2016.1278102

**Published:** 2017-01-09

**Authors:** Henk J. Franssen, Olga Kulikova, Viola Willemsen, Renze Heidstra

**Affiliations:** a Department of Plant Sciences, Laboratory of Molecular Biology, Wageningen University, Wageningen, The Netherlands; b Department of Plant Sciences, Plant Developmental Biology, Wageningen University, Wageningen, The Netherlands

**Keywords:** Cis-regulatory elements, Medicago truncatula, PLETHORA genes, root nodule

## Abstract

Nodules are unique organs formed on roots of legumes by soil-borne bacteria, collectively known as rhizobium. Recently, we have shown that orthologs of the AINTEGUMENTA-like (AIL) AP2 transcription factors PLETHORA (PLT) 1 to 4, that redundantly regulate *Arabidopsis thaliana* root development are involved in root and nodule growth in *Medicago truncatula*. Hence, it is conceivable that rhizobium has co-opted these genes for nodule development. Whether this co-option requires the presence of specific *cis*-elements in the promoters and/or specialization of PLT protein function is not clear. Here, we analyzed the qualitative expression patterns of the Arabidopsis *PLT1* to *4* promoters in Medicago roots and nodules and compared these with the described expression patterns of the Medicago *PLT* genes. Our studies reveal that the expression patterns of the investigated promoters and their Medicago orthologs are very similar, indicating that at least all *cis*-elements regulating spatial *PLT* expression are conserved among the Arabidopsis and Medicago *PLT1 to 4* promoters.

Knowledge of the organization of plant meristems has increased over the last years by studies on model plant species like *Arabidopsis thaliana*. In the root of this model plant, stem cells are surrounding the quiescent center (QC) cells,^1^ which function to prevent stem cell differentiation.^2^ Stem cells and QC form the stem cell niche.^3^ The position of this niche is marked by the highest concentration of PLETHORA (PLT) in a gradient of PLT activity.^4^ PLTs are transcription factors and are part of the small AINTEGUMENTA-like (AIL) AP2 gene clade of transcriptional regulators within the large AP2/ERF family.^5,6^ Among this clade, *PLT1–4* are essential for root formation as their higher order mutants show increasingly severe root phenotypes.^7^

On roots of legumes new organs, so-called root nodules, are formed as a result of the interaction between these plants and soil-borne bacteria collectively known as rhizobia.^8^ In the model legume *Medicago truncatula*, that forms nodules with a persistent meristem at its apex, nodule formation is initiated by dedifferentiation of cortical cells which divide and form the nodule primordium. Specific secreted lipochito-oligosaccharides (Nod factors) allows the rhizobia, through the Nod factor signaling cascade, to control infection and the formation of a nodule meristem at the apex of the primordium.^8,9^ As nodules are formed on roots it has been hypothesized that the nodule developmental program is derived from the lateral root developmental program.^10-15^

Recently, we showed that 4 Medicago *PLT* (*MtPLT1 to 4)* genes redundantly control nodule formation and nodule meristem maintenance.^16^ This is reminiscent of the redundant function of their orthologous *PLT* genes in Arabidopsis root development and lends support to the hypothesis that nodule formation is derived from root developmental programs. Based on our results and that of others it was suggested that rhizobia recruited major regulators of root development.^16-18^ Recruitment may evolve through specialization of protein function^19^ or through specific *cis*-regulatory elements.^20^ In the latter case, it is expected that the Arabidopsis *PLT1* to *4* promoters are not active in the nodule or active at places different from the othologous Medicago *pMtPLT1 to 4* promoters. To determine this, we studied the *pAtPLT1, 2, 3 and 4* promoter mediated expression patterns in Medicago roots and nodules. The length of the promoter regions for *AtPLT* genes used in this study are indicated in Table 1, with a comparison to the *MtPLT* promoters used in Franssen et al., 2015. We constructed transcriptional fusions of the *pAtPLT1, 2, 3 and 4* promoters to the β-glucoronidase (GUS) gene, used *Agrobacterium rhizogenes*-mediated Medicago hairy root transformation^21^ and assayed root and nodule GUS expression.

**Table 1.** Length of Arabidopsis PLT promoters used in this study compared with Medicago PLT promoters.

	Arabidopsis ID	Medicago ID	Length (Kb)^1^	Length (Kb)^2^
PLT1	At3g20840	Medtr2g098180	4.5	1.5
PLT2	At1g51190	Medtr4g065370	1.3	1.3
PLT3	At5g10510	Medtr5g031880	4.6	2.7
PLT4/BBM	At5g17430	Medtr7g080460	4.2	1.1

1 As described in^7^.

2 As described in^16^.

The highest activation of the *pAtPLT::GUS* expression co-localizes with the stem cell niche in Medicago roots (Fig. 1, panels A-D; orange arrow), similar to the patterns observed and described in Arabidopsis roots.^7^ The activation patterns of *pAtPLT3::GUS* and *pAtPLT4::GUS*, however, do not extend in the vasculature as far as the *pMtPLT3::GUS* and *pMtPLT4::GUS* do.^16^ Nevertheless, promoter activation patterns are very similar and therefore the *pAtPLT* promoters must contain conserved *cis*-regulatory elements that are sufficient to drive gene expression in the Medicago root meristem.

**Figure 1.**
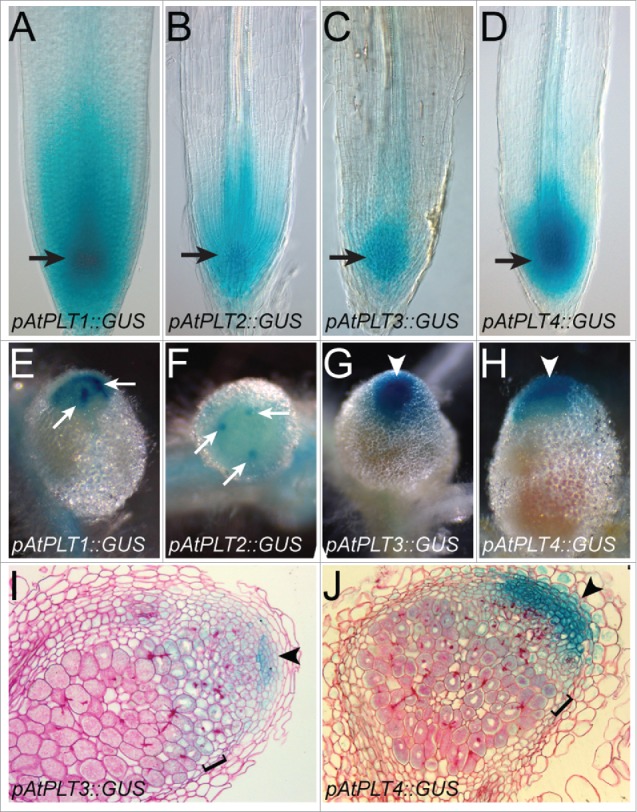
*pAtPLT::GUS* activity in Medicago root and nodule meristem. (A-D) The *pAtPLT1::GUS* (A), *pAtPLT2::GUS* (B), *pAtPLT3::GUS* (C) and *pAtPLT4::GUS* (D) expression patterns overlap in the Medicago root, with the highest activity in the stem cell niche of the root meristem (arrow). (E-F) Top views of *pAtPLT1::GUS* (E) and *pAtPLT2::GUS* (F) nodules show highest GUS activity in discrete regions in the nodule meristem periphery corresponding to the NVM (arrows), and lower GUS activity throughout the nodule meristem. (G-H) *pAtPLT3::GUS* (G) and *pAtPLT4::GUS* (H) display expression throughout the nodule meristem (arrowheads). (I-J) Nodule sections showing highest *pAtPLT3::GUS* (I) and *pAtPLT4::GUS* (J) activity in the central part of the nodule meristem (arrowhead) and lower activity in the infection zone (bracket). Nodules were sampled 16 d after inoculation.

We then studied whether *pAtPLT::GUS* fusions are expressed in the nodule and nodule meristem (Fig. 1E–J). This meristem is composed of a central part, the nodule central meristem (NCM) and of nodule vascular meristems (NVM) that are located at the periphery of the NCM.^16,18^ Whereas *MtPLT1* and *MtPLT2* are expressed highest in the NVM, *MtPLT3* and *MtPLT4* are expressed at equal levels in the NCM and NVM.^16^ Similarly, *pAtPLT1::GUS* and *pAtPLT2::GU*S are highly activated in the NVM (Fig. 1E,F) and the activation patterns of *pAtPLT3::GUS* and *pAtPLT4::GUS* includes both NCM and NVM (Fig. 1G,H). In addition to activation in the NCM and the NVM, expression of *pMtPLT3::GUS* and *pMtPLT4::GUS* was also observed in cells of the infection zone.^16^ Serial sections of *pAtPLT3::GUS* and *pAtPLT4::GUS* nodules were analyzed and these show that also the *pAtPLT3* and *pAtPLT4* promoters are active in the infection zone (Fig. 1I,J). The level of activation of the *pAtPLT3::GUS* and *pAtPLT4::GUS* is lower in the infection zone than in the NM, like for *pMtPL3::GUS* and *pMtPLT4::GUS*.^16^ Thus, in the Medicago nodule the spatial activation of *pAtPLT1 to 4* is similar to that of *pMtPLT1 to 4*. Therefore, all *cis*-regulatory elements for spatial activation in root and specific areas of the nodule must be present in the orthologous Arabidopsis and Medicago *PLT1 to 4* promoters, suggesting that in Medicago no nodule specific *cis*-regularory elements have been acquired to enable the expression of *PLT* genes in nodules.

We show that promoters of *AtPLT1, 2, 3 and 4* genes are activated in Medicago roots in regions previously shown to display the orthologous *MtPLT1 to 4* promoter activity. This indicates that *cis*-elements and the regulatory machinery conferring spatial expression of *PLT1 to 4* in the root meristem are conserved among Arabidopsis and Medicago. Delimiting the activation pattern of *pAtPLT3::GUS* and *pAtPLT4::GUS* in the differentiation zone compared with the more extended vascular expression of *pMtPLT3::GUS* and *pMtPLT4::GUS*, maybe due to differences in promoter sizes tested (Table 1).

Also in the nodule, an organ that cannot be formed on Arabidopsis roots, *pAtPLT1 to 4::GUS* reporters are activated in the same locations as their Medicago orthologues. This indicates that for the spatial activation in nodules all *cis*-elements are present in *AtPLT1 to 4* promoter regions tested here. Based on the similar activation patterns of *At-* and *MtPLT1 to 4* in roots as well as in nodules, it is conceivable that the regulatory mechanisms directing *PLT* expression in these organs share several components.

A next and interesting question is whether the conservation of *PLT* promoter or even PLT protein function between Arabidopsis and Medicago is sufficient for cross complementation. However, single mutants (Arabidopsis) or knock downs (Medicago) display only mild phenotypes.^7,16,22^ Therefore, this should ideally be tested in complementation studies of double mutants in Medicago by *pAtPLT* driven *MtPLT* genes or *pMtPLT* driven *AtPLT* genes.

Our studies indicate that Rhizobium, and by inference the Nod factor signaling cascade, is able to activate *pAtPLT1 to 4* during nodule development. The Arabidopsis genome, however, appears to lack the genetic information for a critical components of this cascade.^23,24^ This raises the question which inputs are essential or sufficient for PLT activation during nodulation. Nod factor application and testing expression in nodulation mutants can be instructive to answer this in Medicago. In parallel, the mechanisms underlying *PLT* expression in Arabidopsis can be useful to further dissect how Rhizobium co-opted genes involved in root development for nodule formation.

Similar to the role of *PLT* genes in Arabidopsis, the Rhizobium-mediated induction of *PLT* gene expression during nodule formation correlates with developing tissues and organs.^6,16^ It is likely that regulated genes/targets of MtPLT proteins will show an overlap with those regulated by AtPLT proteins.^25^ In addition, comparing the MtPLT targets regulated in roots versus nodules may reveal specific *PLT*-mediated developmental programs required for either organ.

## References

[1] Dolan L, Janmaat K, Willemsen V, Linstead P, Poethig S, Roberts K, Scheres B Cellular organization of the *Arabidopsis thaliana* root. Development 1993; 119:71-84; PMID:8275865 8275865 10.1242/dev.119.1.71

[2] van den Berg C, Willemsen V, Hendriks G, Weisbeek P, Scheres B Short-range control of cell differentiation in the Arabidopsis root meristem. Nature 1997; 390:287-9; PMID:9384380; http://dx.doi.org/10.1038/36856 9384380

[3] Scheres B, Lipka V Plant cell biology: get your networks together. Curr Opin Plant Biol 2007; 10(6):546-8; PMID:17964845; http://dx.doi.org/10.1016/j.pbi.2007.09.002 17964845

[4] Mähönen AP, ten Tusscher K, Siligato R, Smetana O, Diaz-Trivino S, Salojarvi J, Wachsman G, Prasad K, Heidstra R, Scheres B PLETHORA gradient formation mechanism separates auxin responses. Nature 2014; 515:125-9; PMID:25156253; http://dx.doi.org/10.1038/nature13663 25156253 PMC4326657

[5] Nole-Wilson S, Tranby TL, Krizek BA AINTEGUMENTA-like (AIL) genes are expressed in young tissues and may specify meristematic or division-competent states. Plant Mol Biol 2005; 57(5):613-28; PMID:15988559; http://dx.doi.org/10.1007/s11103-005-0955-6 15988559

[6] Horstman A, Willemsen V, Boutilier K, Heidstra R AINTEGUMENTA-LIKE proteins: hubs in a plethora of networks. Trend Plant Sci 2014; 19(3):146-57; PMID:24280109; http://dx.doi.org/17960244 10.1016/j.tplants.2013.10.010 24280109

[7] Galinha C, Hofhuis H, Luijten M, Willemsen V, Blilou I, Heidstra R, Scheres B PLETHORA proteins as dose-dependent master regulators of Arabidopsis root development. Nature 2007; 449:1053-7; PMID:17960244; http://dx.doi.org/10.1038/nature06206 17960244

[8] Suzaki T, Yoro E, Kawaguchi M Leguminous plants: inventors of root nodules to accommodate symbiotic bacteria. Inter Rev Cell Mol Biol 2015; 316:111-58; PMID:25805123; http://dx.doi.org/10.1016/bs.ircmb.2015.01.004 25805123

[9] Xiao TT, Schilderink S, Moling S, Deinum EE, Kondorosi E, Franssen H, Kulikova O, Niebel A, Bisseling T Fate map of *Medicago truncatula* root nodules. Development 2014; 141:3517-28; PMID:25183870; http://dx.doi.org/10.1242/dev.110775 25183870

[10] Nutman PS Physiological studies on nodule formation.1. The relation between nodulation and lateral root formation in red clover. Annal Botany 1948; 12:81-96

[11] Hirsch AM, La Rue TA, Doyle J Is the legume nodule a modified root or stem or an organ sui generis? Crit Rev Plant Sci 1997; 16:361-92; http://dx.doi.org/10.1080/07352689709701954

[12] Mathesius U, Weinman JJ, Rolfe BG, Djordjevic MA Rhizobia can induce nodules in white clover by “hijacking” mature cortical cells activated during lateral root development. Mol Plant Microbe Interact 2000; 13:170-82; PMID:10659707; http://dx.doi.org/10.1094/MPMI.2000.13.2.170 10659707

[13] de Billy F, Grosjean C, May S, Bennett M, Cullimore JV Expression studies on AUX1-like genes in *Medicago truncatula* suggest that auxin is required at two steps in early nodule development. Mol Plant Microb Interact 2001; 14:267-77; PMID:11277424; http://dx.doi.org/10.1094/MPMI.2001.14.3.267 11277424

[14] Bright LJ, Liang Y, Mitchell DM, Harris JM The LATD gene of *Medicago truncatula* is required for both nodule and root development. Mol Plant Microb Interact 2005; 18:521-32; PMID:15986921; http://dx.doi.org/10.1094/MPMI-18-0521 15986921

[15] Desbrosses GJ, Stougaard J Root nodulation: a paradigm for how plant-microbe symbiosis influences host developmental pathways. Cell Host Microbe 2011; 10:348-58; PMID:22018235; http://dx.doi.org/26253408 10.1016/j.chom.2011.09.005 22018235

[16] Franssen H, Xiao TT, Kulikova O, Wan X, Bisseling T, Scheres B, Heidstra R Root developmental programs shape *Medicago truncatula* nodule meristem. Development 2015; 142:2941-50; PMID:26253408; http://dx.doi.org/10.1242/dev.120774 26253408

[17] Osipova MA, Mortier V, Demchenko KN, Tsyganov VE, Tikhonovich IA, Lutova LA, Dolgikh EA, Goormachtig S WUSCHEL-RELATED HOMEOBOX5 gene expression and interaction of CLE peptides with components of the systemic control add two pieces to the puzzle of autoregulation of nodulation. Plant Physiol 2012; 158:1329-41; PMID:22232385; http://dx.doi.org/10.1104/pp.111.188078 22232385 PMC3291250

[18] Roux B, Rodde N, Jardinaud MF, Timmers T, Sauviac L, Cottret L, Carrere S, Sallet E, Courcelle E, Moreau S, et al. An integrated analysis of plant and bacterial gene expression in symbiotic root nodules using laser-capture microdissection coupled to RNA sequencing. Plant J 2014; 77:817-37; PMID:24483147; http://dx.doi.org/10.1111/tpj.12442 24483147

[19] Shubin N, Tabin C, Carroll S Deep homology and the origins of evolutionary novelty. Nature 2009; 457:818-23; PMID:19212399; http://dx.doi.org/10.1038/nature07891 19212399

[20] Rebeiz M, Jikomes N, Kassner VA, Carroll SB Evolutionary origin of a novel gene expression pattern through co-option of the latent activities of existing regulatory sequences. Proc Natl Acad Sci U S A 2011; 108:10036-43; PMID:21593416; http://dx.doi.org/10.1073/pnas.1105937108 21593416 PMC3121811

[21] Limpens E, Ramos J, Franken C, Raz V, Compaan B, Franssen H, Bisseling T, Geurts R RNA interference in Agrobacterium rhizogenes-transformed roots of Arabidopsis and Medicago truncatula. J Exp Bot 2004; 55:983-92; PMID:15073217; http://dx.doi.org/10.1093/jxb/erh122 15073217

[22] Aida M, Beis D, Heidstra R, Willemsen V, Blilou I, Galinha C, Nussaume L, Noh YS, Amasino R, Scheres B The PLETHORA genes mediate patterning of the Arabidopsis root stem cell niche. Cell 2004; 119(1):109-20; PMID:15454085; http://dx.doi.org/10.1016/j.cell.2004.09.018 15454085

[23] Delaux PM, Varalaz K, Edgers PP, Coruzzi GM, Pires JC, Ane JM Comparative phylogenomics uncover the impact of symbiotic associations on host genome evolution. PLoS Genetics 2014; 10(7):1-12; PMID:25032823; http://dx.doi.org/10.1371/journal.pgen.1004487 PMC4102449 25032823

[24] Bravo A, York T, Pumplin N, Mueller LA, Harrison MJ Genes conserved for arbuscular mycorrhizal symbiosis identified through phylogenomics. Nat Plant 2016; 2:1-6; PMID:27249190; http://dx.doi.org/10.1038/nplants.2015.208 27249190

[25] Santuari L, Sanchez-Perez G, Luijten M, Rutjens B, Terpstra I, Berke L, Gorte M, Prasad K, Bao D, Timmermans-Hereijgers JL, et al. The PLETHORA gene regulatory network guides growth and cell differentiation in Arabidopsis roots. Plant Cell 2016; (28):2937-51; PMID:27920338; http://dx.doi.org/10.1105/tpc.16.00656 PMC5240741 27920338

